# Supplementary material for: Generation and optimization of off-the-shelf immunotherapeutics targeting TCR-Vβ2+ T cell malignancy
Source: Nat Commun. 2024 Jan 15;15:519. doi: 10.1038/s41467-024-44786-2 (PMC10789731; doi:10.1038/s41467-024-44786-2)
Supplement: Supplementary file 1 — Supplementary Information [file 41467_2024_44786_MOESM1_ESM.pdf]

Title: Generation and optimization of off-the-shelf immunotherapeutics targeting TCR-Vβ2+ T cell malignancy

Jingjing Ren[1,*] †, Xiaofeng Liao[1,* †], Julia M. Lewis[1], Jungsoo Chang[1], Rihao Qu[2], Kacie R. Carlson[1], Francine Foss[3], Michael Girardi[1,†]

[1]Department of Dermatology, Yale School of Medicine, New Haven, CT, USA.

[2] The Computational Biology and Bioinformatics Program, Yale School of Medicine, New Haven, CT, USA.

[3]Department of Internal Medicine, Section of Medical Oncology, Yale School of Medicine, New Haven, CT, USA.

* These authors contributed equally.

† Co-corresponding authors:

Michael Girardi, MD; Email: michael.girardi@yale.edu

Jingjing Ren, MD, PhD; Email: jingjing.ren@yale.edu

Xiaofeng Liao, PhD; Email: xiaofeng.liao@yale.edu

Supplementary Fig. 1: mCAR-Vβ2 T cell development.

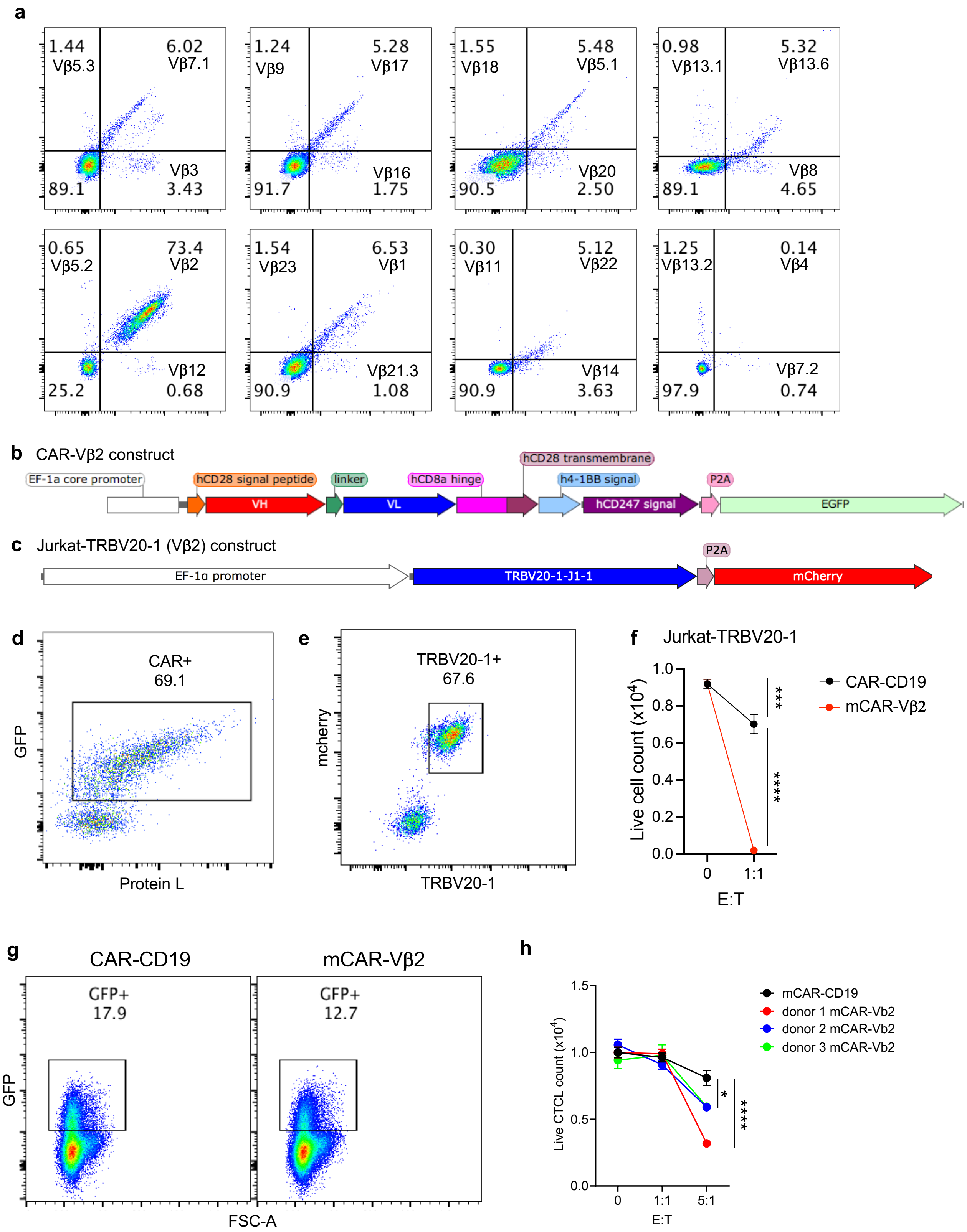

Supplementary Fig. 1: mCAR-Vβ2 T cell development.

a, Representative flow cytometry of total T cells from a CTCL patient with a Vβ2+ malignancy stained with a panel of anti-Vβ antibodies showing the distribution of Vβ family usage and predominance of Vβ2. **b,** Key components of CAR-Vβ2 construction in a lentiviral vector. **c,** Key components of TCR-β chain TRBV20-1 containing construction in a lentiviral vector. **d,** GFP and surface CAR-Vβ2 expression in mCAR-Vβ2 T cells determined by flow cytometry. **e,** mcherry and surface TRBV20-1 expression in Jurkat-TRBV20-1 cells determined by flow cytometry. **f,** Live Jurkat-TRBV20-1 cell counts after overnight in vitro killing by allogeneic CAR-CD19 T cells (black) or mCAR-Vβ2 T cells (red) at 1:1 of E:T ratio, determined by flow cytometry. **g,** Lentiviral transduction efficiency of CAR-CD19 or mCAR-Vβ2 in CD8 T cells from a Vβ2+ CTCL patient, determined by flow cytometry detection of GFP. **h,** Live CTCL counts of three Vβ2+ CTCL patients after overnight in vitro killing by purified autologous CAR-CD19 T cells (black) or mCAR-Vβ2 T cells (red, blue and green) at different E:T ratios, determined by flow cytometry. Source data are provided as a Source Data file.

Supplementary Fig. 2: Allogeneic mCAR-Vβ2 T cell generation.

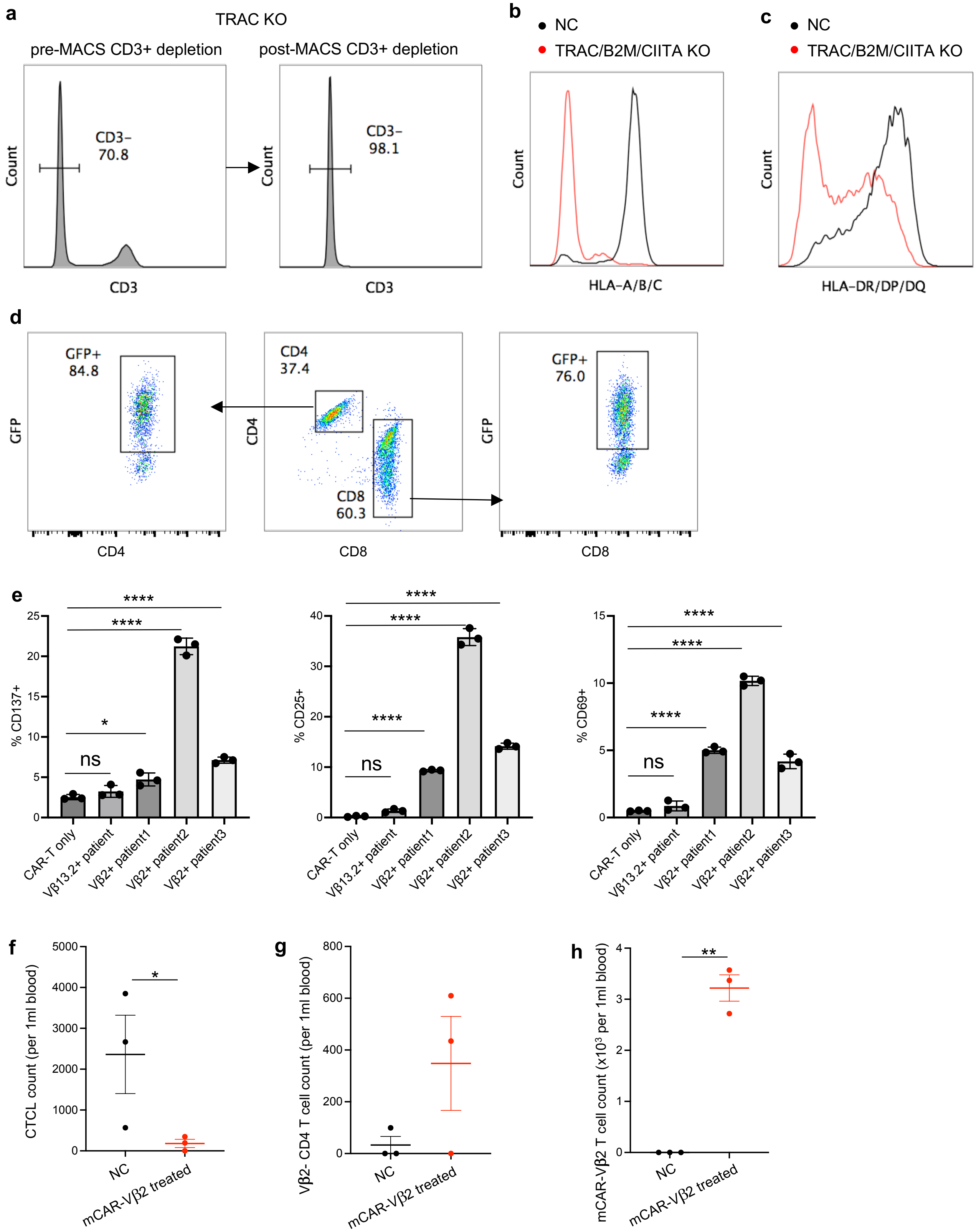

Supplementary Fig. 2: Allogeneic mCAR-Vβ2 T cell generation.

a, Flow cytometric histograms showing CD3 expression 2 days post TRAC KO in mCAR-Vβ2 T cells before (left) or after (right) residual CD3+ cell depletion. **b,** HLA-A/B/C and **c,** HLA-DR/DP/DQ expression on mCAR-Vβ2 T cells with (red) or without (normal control (NC), black) triple (TRAC/B2M/CIITA) KO, determined by flow cytometry. **d,** Lentiviral transduction efficiency of mCAR-Vβ2 in CD8 and CD4 T cells from a healthy donor following triple KO, determined by flow cytometry detection of GFP. **e,** Activation marker CD137, CD25 and CD69 expression on triple KO mCAR-Vβ2 T cells after overnight in vitro culture alone or mixed with CTCL cells from three different Vβ2+ patients and one Vβ13.2+ patient, determined by flow cytometry. **f,** Vβ2+ PTCL cell count, **g,** Vβ2-normal CD4+ T cell count, and **h,** mCAR-Vβ2 T cell count in 1ml blood from NSG mice carrying CD4+ malignant T cells from a Vβ2+ PTCL patient and treated with (red) or without (NC, black) triple KO mCAR-Vβ2 T cells. **e,** n=3 replicates of each group. *$p<0.05$ and ****$p<0.0001$ by one-way ANOVA. **f-h,** n=3 mice in each group (**f,** p=0.032, **h,** p=0.0026). *$p<0.05$ and **$p<0.01$ by t-test. Source data are provided as a Source Data file.

Supplementary Fig. 3: CAR expression levels in humanized CAR-Vβ2 T cells.

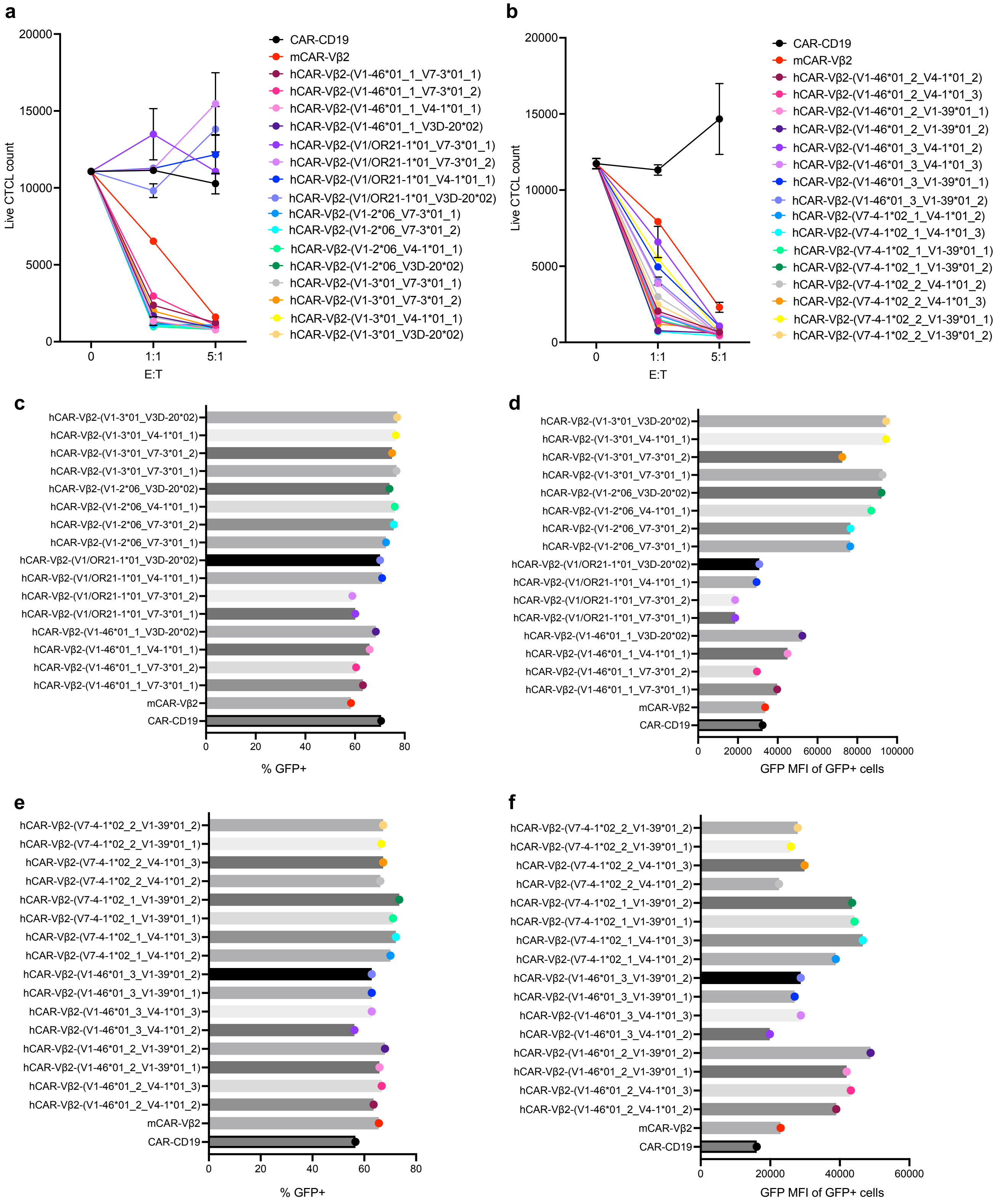

Supplementary Fig. 3: CAR expression levels in humanized CAR-Vβ2 T cells.

a-b, Live counts of CTCL cells from a Vβ2+ patient after overnight culture with a set of CAR-Vβ2 T cells humanized via two different in silico strategies provided by **a,** the BioPhi algorithm or **b,** a third-party contractor (mAbvice), as determined by flow cytometry. c**,** GFP+% and d**,** average GFP expression intensity of the GFP+ population of CAR-Vβ2 T cells after humanization using the BioPhi in silico algorithm, as determined by flow cytometry. e**,** GFP+% and f**,** average GFP expression intensity of the GFP+ population of CAR-Vβ2 T cells after humanization by mAbvice, as determined by flow cytometry. Source data are provided as a Source Data file.

Supplementary Fig. 4: Immune reactivity of candidate humanized CAR-Vβ2.

a

hCAR-Vβ2-(V1-46*01_2_V4-1*01_2)	hCAR-Vβ2-(V1-46*01_2_V4-1*01_3)	hCAR-Vβ2-(V1-46*01_2_V1-39*01_1)	hCAR-Vβ2-(V1-46*01_2_V1-39*01_2)	hCAR-Vβ2-(V1-46*01_3_V4-1*01_2)	hCAR-Vβ2-(V1-46*01_3_V4-1*01_3)	hCAR-Vβ2-(V1-46*01_3_V1-39*01_1)	hCAR-Vβ2-(V1-46*01_3_V1-39*01_2)	hCAR-Vβ2-(V7-4-1*02_1_V4-1*01_2)
hCAR-Vβ2-(V7-4-1*02_1_V4-1*01_3)	hCAR-Vβ2-(V7-4-1*02_1_V1-39*01_1)	hCAR-Vβ2-(V7-4-1*02_1_V1-39*01_2)	hCAR-Vβ2-(V7-4-1*02_2_V4-1*01_2)	hCAR-Vβ2-(V7-4-1*02_2_V4-1*01_3)	hCAR-Vβ2-(V7-4-1*02_2_V1-39*01_1)	hCAR-Vβ2-(V7-4-1*02_2_V1-39*01_2)	CAR-CD19	mCAR-Vβ2
hCAR-Vβ2-(V1-46*01_1_V7-3*01_1)	hCAR-Vβ2-(V1-46*01_1_V7-3*01_2)	hCAR-Vβ2-(V1-46*01_1_V4-1*01_1)	hCAR-Vβ2-(V1-46*01_1_V3D-20*02)	hCAR-Vβ2-(V1/OR21-1*01_V7-3*01_1)	hCAR-Vβ2-(V1/OR21-1*01_V7-3*01_2)	hCAR-Vβ2-(V1/OR21-1*01_V4-1*01_1)	hCAR-Vβ2-(V1/OR21-1*01_V3D-20*02)	hCAR-Vβ2-(V1-2*06_V7-3*01_1)
hCAR-Vβ2-(V1-2*06_V7-3*01_2)	hCAR-Vβ2-(V1-2*06_V4-1*01_1)	hCAR-Vβ2-(V1-2*06_V3D-20*02)	hCAR-Vβ2-(V1-3*01_V7-3*01_1)	hCAR-Vβ2-(V1-3*01_V7-3*01_2)	hCAR-Vβ2-(V1-3*01_V4-1*01_1)	hCAR-Vβ2-(V1-3*01_V3D-20*02)	CAR-CD19	mCAR-Vβ2

b

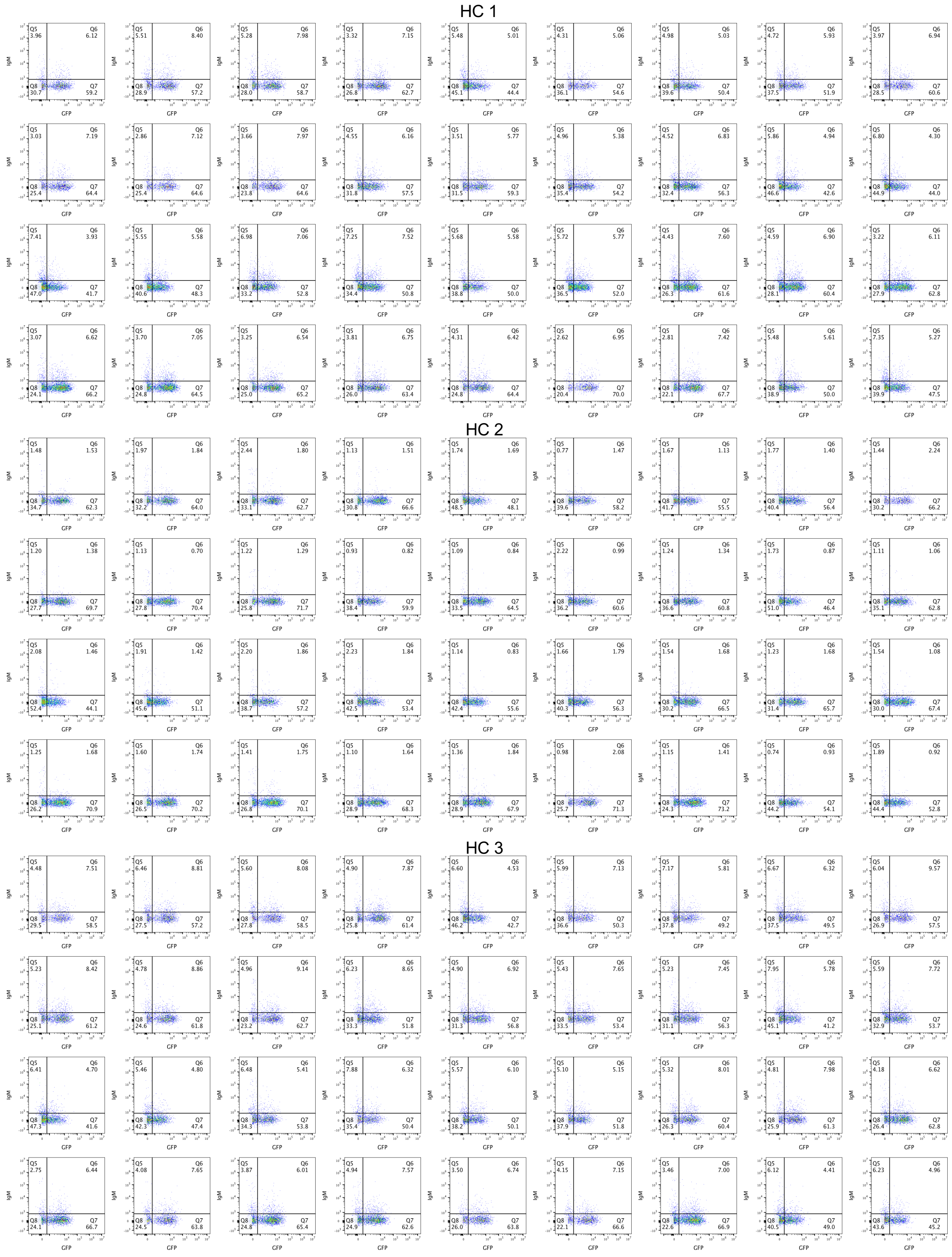

c

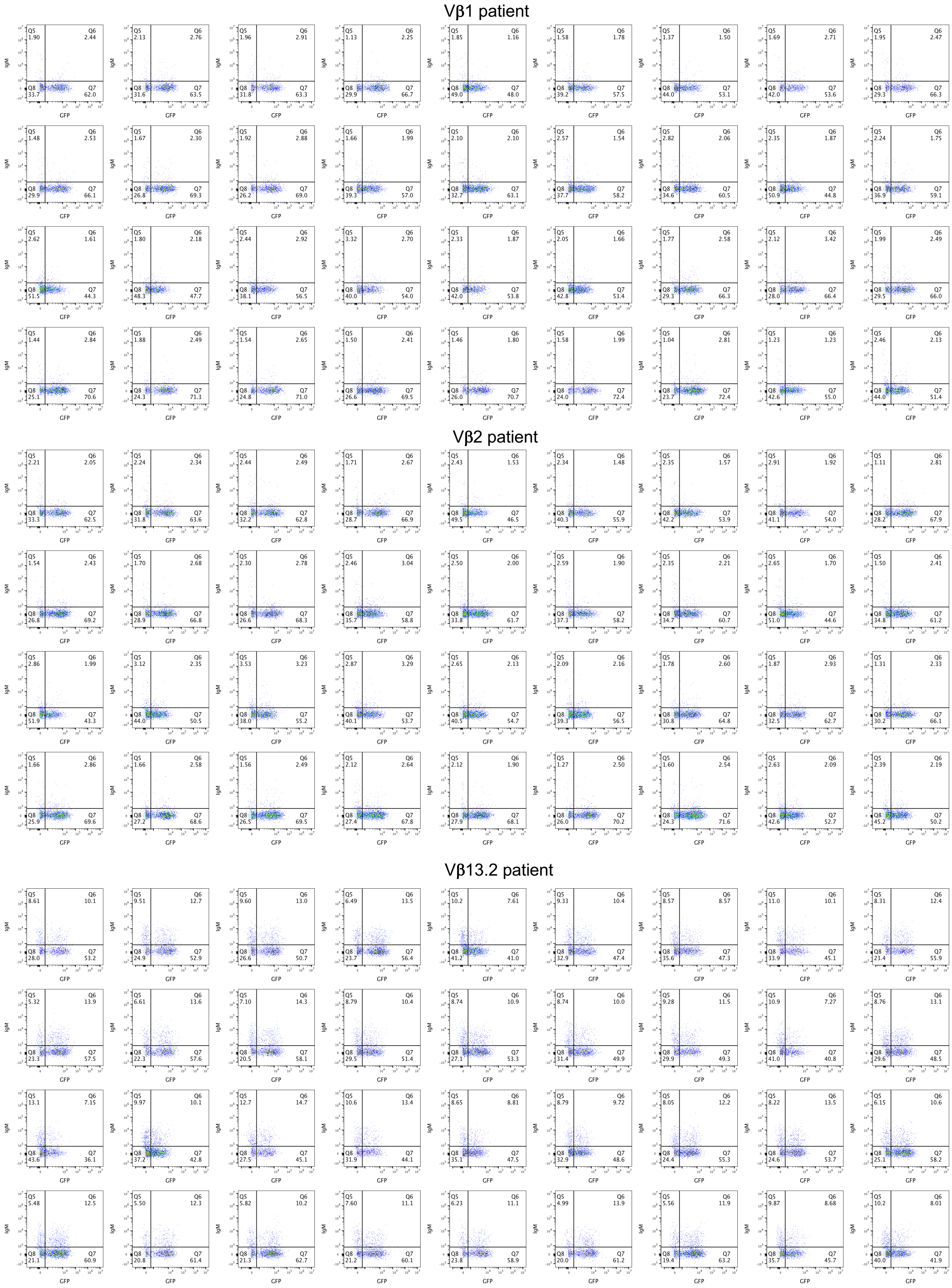

d

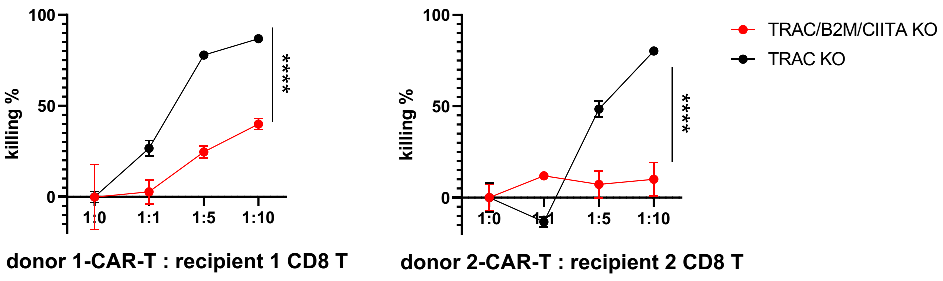

Supplementary Fig. 4: Immune reactivity of candidate humanized CAR-Vβ2.

a, The sample layout used to test for the presence of pre-existing IgM antibody directed against the panel of allogeneic humanized CAR-Vβ2 T cells in sera from **b,** three healthy controls or **c,** three CTCL patients, as determined by flow cytometry. **d,** MLR assay to detect killing by CD8 T cells from two donors mixed with TRAC-KO (black) or TRAC/B2M/CIITA (triple)-KO hCAR-Vβ2 T cells generated from two additional donors. n=3 replicates of each group. ****p<0.0001 by two-way ANOVA. Source data are provided as a Source Data file.

Supplementary Fig. 5: CRISPR-AAV system for CAR-Vβ2 T cell generation.

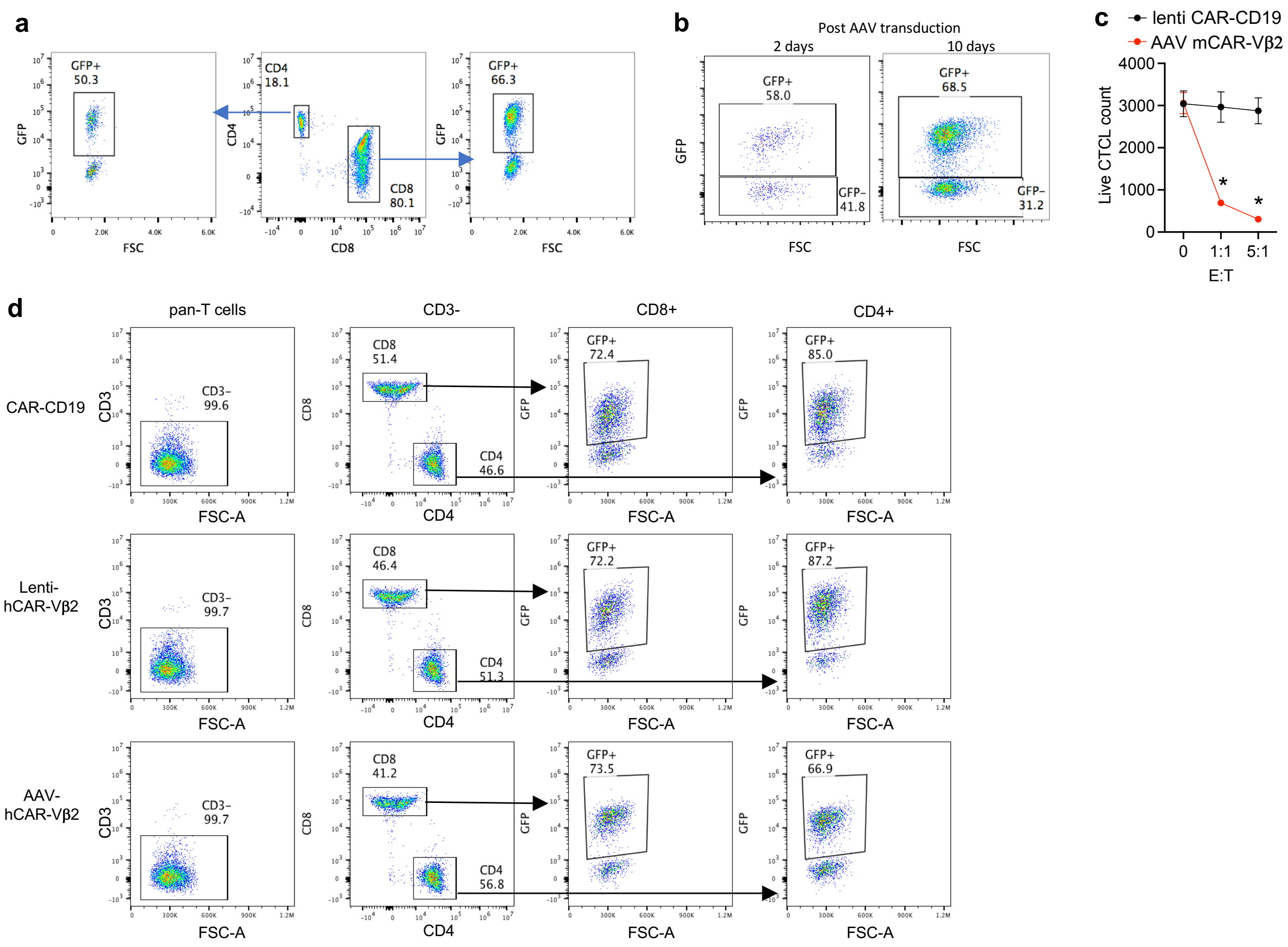

Supplementary Fig. 5: CRISPR-AAV system for CAR-Vβ2 T cell generation.

a, Representative flow cytometry showing CD4 and CD8 population percentages and expression of GFP as CAR reporter on AAV-dependent allogeneic mCAR-Vβ2 T cells. **b,** Expression of GFP as CAR reporter on mCAR-Vβ2 T cells two days and ten days post triple KO and AAV-mCAR-Vβ2 transduction, determined by flow cytometry. **c,** Patient derived Vβ2+ CTCL cell counts after overnight in vitro co-culture with allogeneic triple KO mCAR-Vβ2 pan-T cells generated via CRISPR/AAV system (red) compared to allogeneic triple KO CAR-CD19 pan-T cells generated via lentivirus transduction (black), at different effector to target (E:T) ratios. **d,** Representative flow cytometry showing CD3- purity, CD4 and CD8 population percentages and expression of GFP as CAR reporter on lenti-CAR-CD19, lenti-hCAR-Vβ2 and AAV-hCAR-Vβ2 T cells. **c,** n=3 replicates in each group. *$p<0.05$ by two-way ANOVA. Source data are provided as a Source Data file.

Supplementary Fig. 6: hCAR-Vβ2 T cell recovery in cytokine optimization.

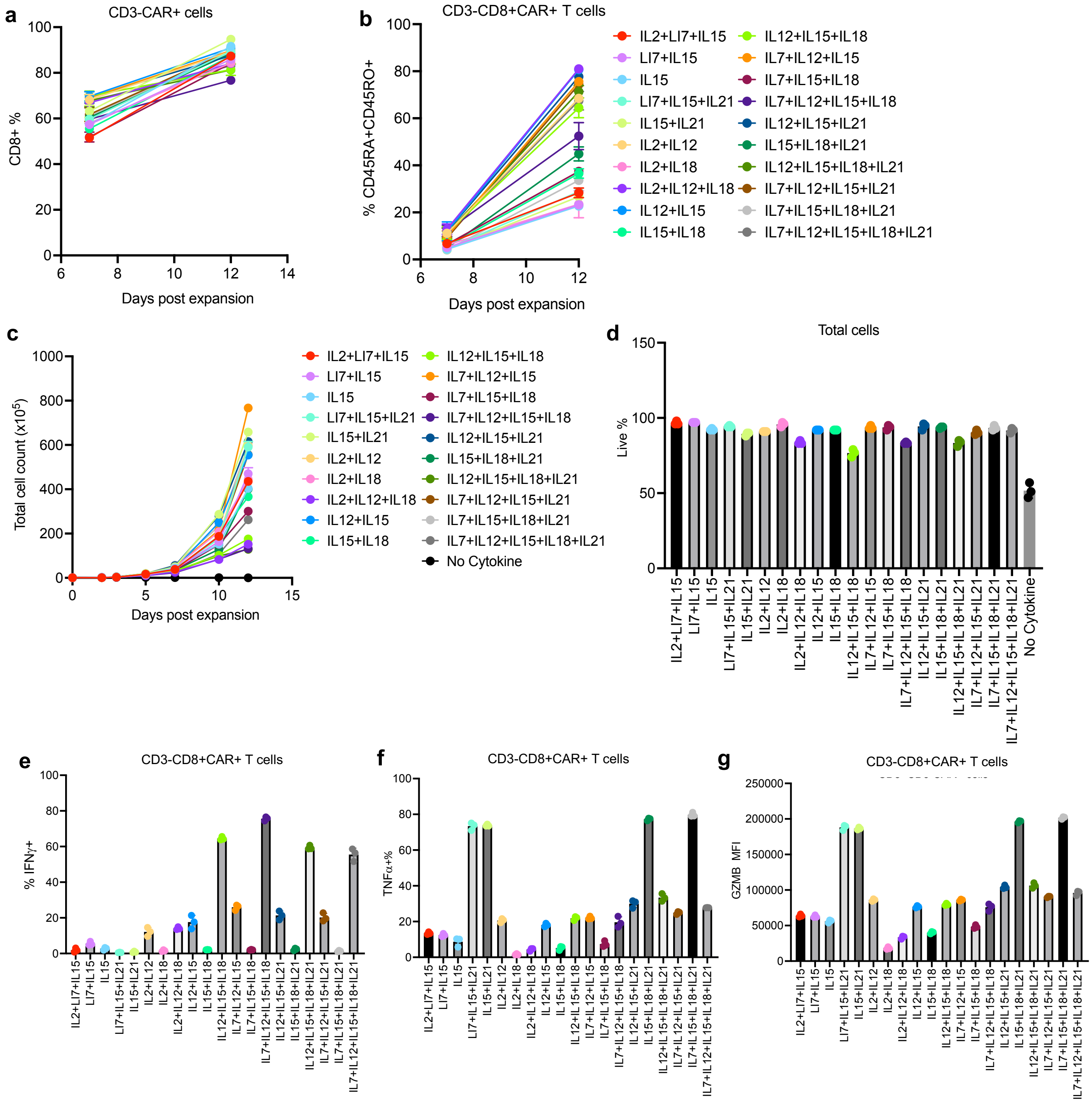

Supplementary Fig. 6: hCAR-Vβ2 T cell recovery in cytokine optimization.

a, CD8+ % in 7-day and 12-day cytokine expanded CD3- hCAR-Vβ2 T cells, determined by flow cytometry. n=3 replicates in each group. **b,** CD45RA+CD45RO+ % of 7-day and 12-day cytokine expanded resting CD8+CD3- hCAR-Vβ2 T cells, determined by flow cytometry. **c,** Live total T cell counts during cytokine expansion in vitro, determined by trypan blue cell counting. **d,** Percentage of viable total T cells following 12-day cytokine expansion, determined by trypan blue cell counting. **e,** IFNγ+ % of 12-day cytokine expanded resting CD8+CD3- hCAR-Vβ2 T cells, determined by flow cytometry. **f,** TNFα+ % and **g,** GZMB MFI of 12-day cytokine expanded resting CD8+C3- hCAR-Vβ2 T cells, determined by flow cytometry. Source data are provided as a Source Data file.

Supplementary Fig. 7: Humanized anti-Vβ2 therapeutic antibody with enhanced ADCC.

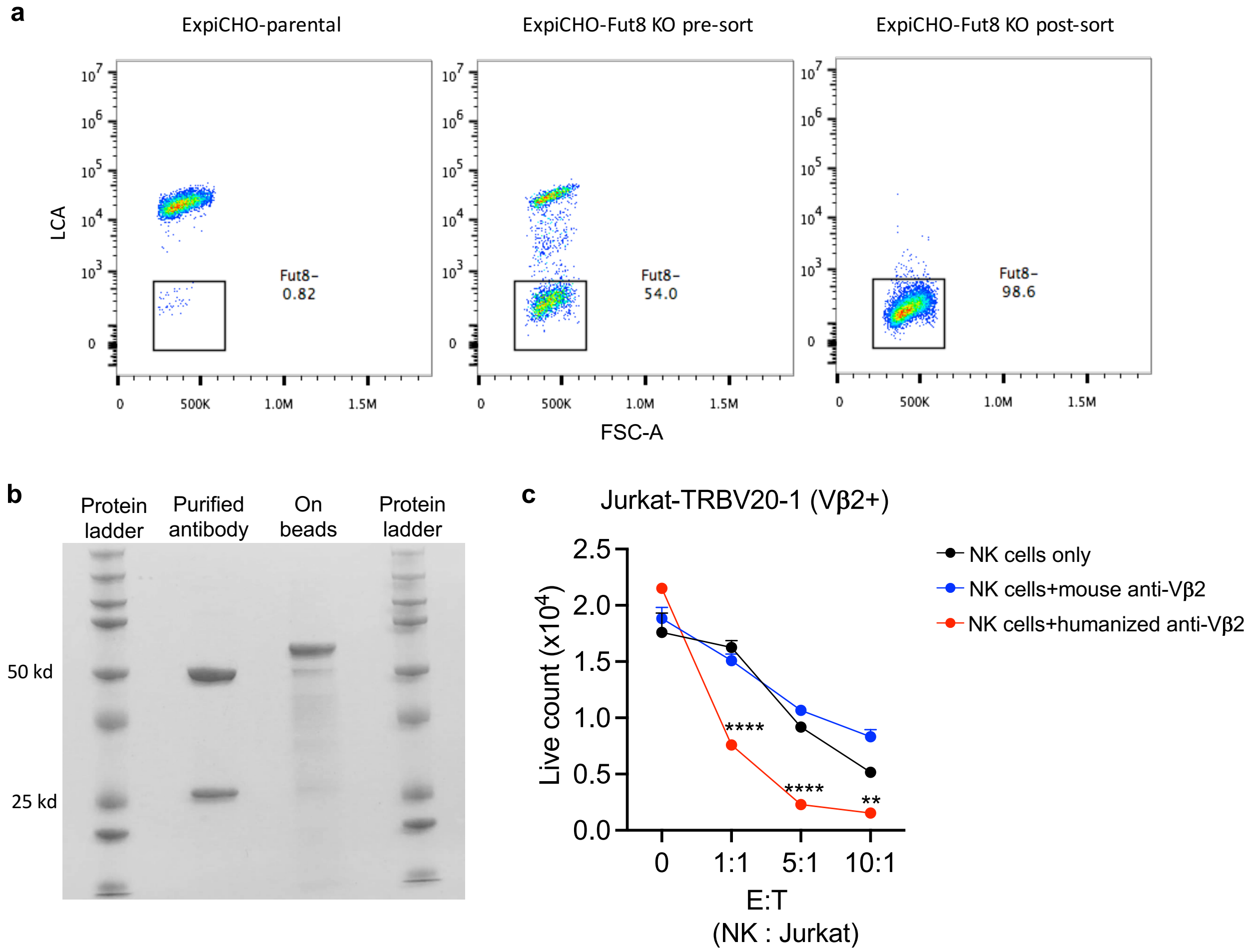

Supplementary Fig. 7: Humanized anti-Vβ2 therapeutic antibody with enhanced ADCC.

a, Lens Culinaris Agglutinin (LCA) expression on the surface of parental expiCHO cells, pre-sorted expiCHO cells post Fut8 KO or post-sorted expiCHO cells post Fut8 KO, determined by flow cytometry. **b,** SDS-PAGE of purified humanized anti-Vβ2 antibody sample and denatured protein sample from protein G beads post antibody elution. **c,** Live Jurkat-TRBV20-1 cell counts, after overnight co-culture with NK cells from a healthy donor as effector cells at different E:T ratios without antibody addition (black) or mixed with 100ng/ml mouse anti-Vβ2 antibody (blue) or humanized anti-Vβ2 antibody (red), determined by flow cytometry. n=3 replicates in each group. **$p<0.01$ and ****$p<0.0001$ by two-way ANOVA. Source data are provided as a Source Data file.

Supplementary Fig. 8: Flow cytometry gating strategies.

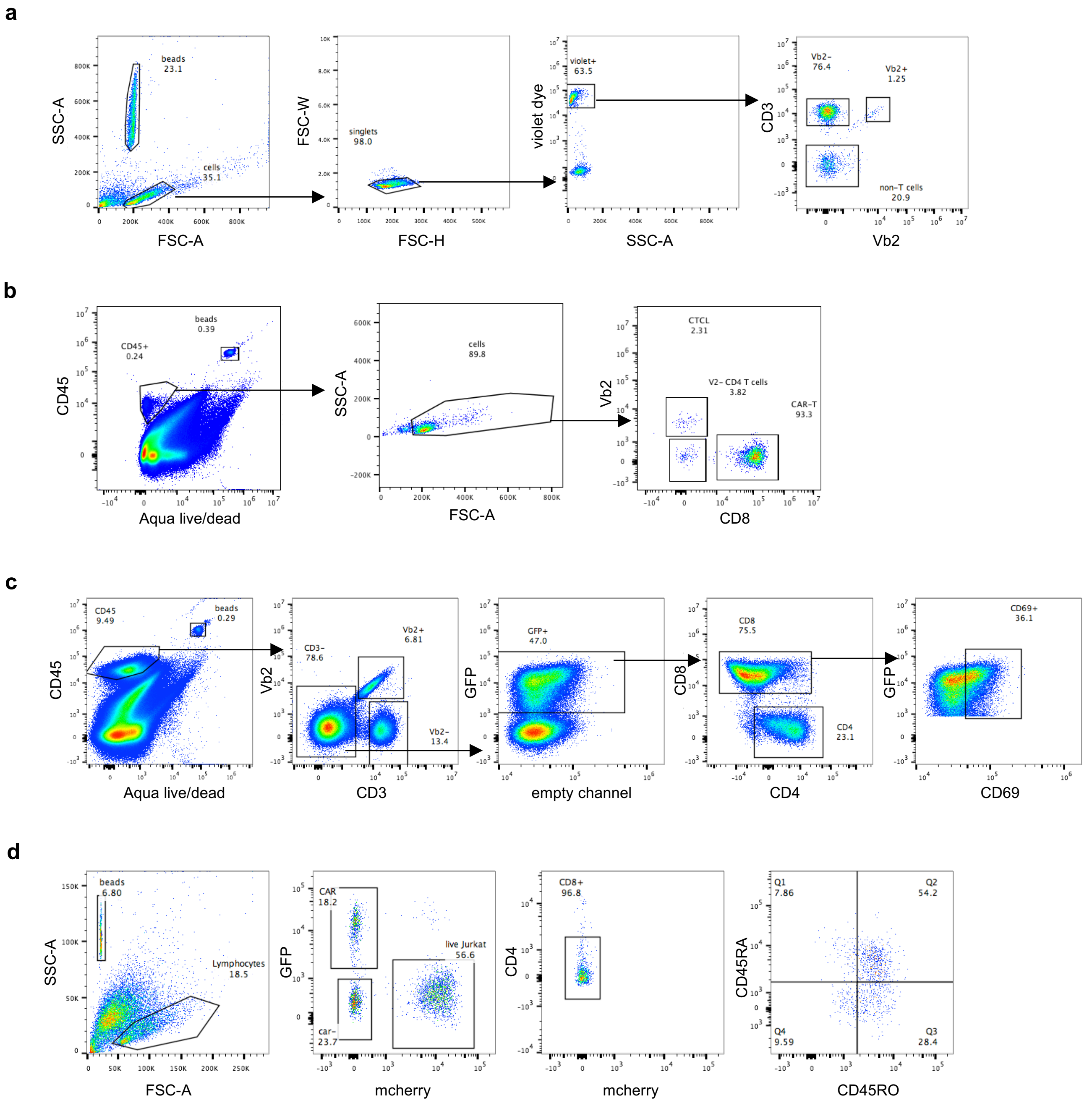

Supplementary Fig. 8: Flow cytometry gating strategies.

a, Gating strategy for Fig. 1 c-f, Fig. 4 c-d and Fig. 6 c-h. b, Gating strategy for Fig. 2 b-l and Fig. 3. c, Gating strategy for Fig. 4 e-k. d, Gating strategy for Fig. 5 c-f.

Supplementary Table 1.

No.	cytokine combination
1	IL2+IL7+IL15
2	IL7+IL15
3	IL15
4	IL7+IL15+IL21
5	IL15+IL21
6	IL2+IL12
7	IL2+IL18
8	IL2+IL12+IL18
9	IL12+IL15
10	IL15+IL18
11	IL12+IL15+IL18
12	IL7+IL12+IL15
13	IL7+IL15+IL18
14	IL7+IL12+IL15+IL18
15	IL12+IL15+IL21
16	IL15+IL18+IL21
17	IL12+IL15+IL18+Il21
18	IL7+IL12+IL15+IL21
19	IL7+IL15+IL18+IL21
20	IL7+IL12+IL15+IL18+IL21

Supplementary Table 2.

Gene	sgRNA sequence
TRAC	TCAGGGTTCTGGATATCTGT
B2M	AAGTCAACTTCAATGTCGGA
CIITA	GTGGCACACTGTGAGCTGCC
TRBV12-3	GCAAAGGGACACACAGCAGA
Fut8	AGTTGAAACTCTGAAAATGC
