# Supplementary material for: Generation and optimization of off-the-shelf immunotherapeutics targeting TCR-Vβ2+ T cell malignancy
Source: Nat Commun. 2024 Jan 15;15:519. doi: 10.1038/s41467-024-44786-2 (PMC10789731; doi:10.1038/s41467-024-44786-2)
Supplement: Supplementary file 3 — Reporting Summary [file 41467_2024_44786_MOESM3_ESM.pdf]

Corresponding author(s): Michael Girardi, Jingjing Ren, Xiaofeng Liao

Last updated by author(s): Dec 10, 2023

# Reporting Summary

Nature Portfolio wishes to improve the reproducibility of the work that we publish. This form provides structure for consistency and transparency in reporting. For further information on Nature Portfolio policies, see our Editorial Policies and the Editorial Policy Checklist.

## Statistics

For all statistical analyses, confirm that the following items are present in the figure legend, table legend, main text, or Methods section.

| n/a | Confirmed | |
|---|---|---|
| ☐ | ☒ | The exact sample size ($n$) for each experimental group/condition, given as a discrete number and unit of measurement |
| ☐ | ☒ | A statement on whether measurements were taken from distinct samples or whether the same sample was measured repeatedly |
| ☐ | ☒ | The statistical test(s) used AND whether they are one- or two-sided<br>*Only common tests should be described solely by name; describe more complex techniques in the Methods section.* |
| ☐ | ☒ | A description of all covariates tested |
| ☒ | ☐ | A description of any assumptions or corrections, such as tests of normality and adjustment for multiple comparisons |
| ☐ | ☒ | A full description of the statistical parameters including central tendency (e.g. means) or other basic estimates (e.g. regression coefficient) AND variation (e.g. standard deviation) or associated estimates of uncertainty (e.g. confidence intervals) |
| ☐ | ☒ | For null hypothesis testing, the test statistic (e.g. $F$, $t$, $r$) with confidence intervals, effect sizes, degrees of freedom and $P$ value noted<br>*Give P values as exact values whenever suitable.* |
| ☒ | ☐ | For Bayesian analysis, information on the choice of priors and Markov chain Monte Carlo settings |
| ☒ | ☐ | For hierarchical and complex designs, identification of the appropriate level for tests and full reporting of outcomes |
| ☒ | ☐ | Estimates of effect sizes (e.g. Cohen's $d$, Pearson's $r$), indicating how they were calculated |

*Our web collection on statistics for biologists contains articles on many of the points above.*

## Software and code

Policy information about availability of computer code

| | |
|---|---|
| Data collection | Cytoflex CytExpert, BioPhi, IVIS Lumina X5 |
| Data analysis | GraphPad Prism9, FlowJo v.10, Living Image® Software v4.8.0 (IVIS Imaging Systems) |

For manuscripts utilizing custom algorithms or software that are central to the research but not yet described in published literature, software must be made available to editors and reviewers. We strongly encourage code deposition in a community repository (e.g. GitHub). See the Nature Portfolio guidelines for submitting code & software for further information.

## Data

Policy information about availability of data

All manuscripts must include a data availability statement. This statement should provide the following information, where applicable:

- Accession codes, unique identifiers, or web links for publicly available datasets
- A description of any restrictions on data availability
- For clinical datasets or third party data, please ensure that the statement adheres to our policy

Data supporting the findings of the study are available within the Article, Supplementary Information and Source data file. Human-related data that were collected but not shown in the paper might be subject to confidentiality (e.g., sex and age). All outstanding data are available upon request from the corresponding author, Michael Girardi (michael.girardi@yale.edu) due to the intellectual property protection applications that are currently under consideration for the disclosed

innovations.

## Research involving human participants, their data, or biological material

Policy information about studies with human participants or human data. See also policy information about sex, gender (identity/presentation), and sexual orientation and race, ethnicity and racism.

| | |
|---|---|
| Reporting on sex and gender | These findings are not sex or gender specific. Individuals of both sexes, regardless of gender, were invited to participate in the study. Sex assigned at birth from their medical record is used for reporting. Low sample size precludes sex- and gender-based analyses. |
| Reporting on race, ethnicity, or other socially relevant groupings | These findings are not race, ethnicity or other socially relevant groupings specific. |
| Population characteristics | Patients previously diagnosed with CTCL or PTCL who have an identifiable malignant T cell population in their peripheral blood will be recruited. This includes both males and females of all races ranging in age from 18-90 years. Because disease incidence increases with increasing age, older subjects predominate. In this study, a limited number of anonymous adult volunteers aged from 35-55 were recruited as healthy donors without a history of cancer or immune disorders. |
| Recruitment | YNHH Oncology and Photopheresis Unit physicians or mid-level provider discuss the possibility of participating in this study with their patients during the normal course of their care for the treatment of CTCL or PTCL. We impartially chose patients with a significant presence of high blood cancer involvement. The healthy donors in this study were randomly selected from available volunteers. Informed consent was obtained. |
| Ethics oversight | The protocols involved in this study are all approved by Yale University Institutional Review Board and Human Investigation Committee |

Note that full information on the approval of the study protocol must also be provided in the manuscript.

# Field-specific reporting

Please select the one below that is the best fit for your research. If you are not sure, read the appropriate sections before making your selection.

☒ Life sciences ☐ Behavioural & social sciences ☐ Ecological, evolutionary & environmental sciences

For a reference copy of the document with all sections, see nature.com/documents/nr-reporting-summary-flat.pdf

# Life sciences study design

All studies must disclose on these points even when the disclosure is negative.

| | |
|---|---|
| Sample size | Sample sizes were primarily determined by availability of materials, e.g. patient-derived malignant cells. No power calculations were performed. The sample size for in vivo mouse studies utilizing the Jurkat-Vb2 tumor cell line were determined based on pilot experiments assessing the tumor cell line growth characteristics which we found to be sufficiently similar from mouse to mouse such that groups of N=5 mice could be used to reasonably assess the effects of different treatments. |
| Data exclusions | No data were excluded from the analyses. |
| Replication | To ensure robustness of the data two different viral vectors (AAV and Lentivirus) were used and in this way CAR-T manufacturing was replicated. Both Vb2 expressing Jurkat cells and Vb2+ patient derived cells were used as a way to replicate both in vitro and in vivo experiments. Experiments were also repeated by using cells from multiple donors. All attempts at replication were successful. |
| Randomization | Tumor-bearing mice were randomly allocated into experimental groups. Cells from limited donors were equally used into different experimental conditions for in vitro studies. |
| Blinding | Investigators were blinded to group identification for data collection and analysis. |

# Reporting for specific materials, systems and methods

We require information from authors about some types of materials, experimental systems and methods used in many studies. Here, indicate whether each material, system or method listed is relevant to your study. If you are not sure if a list item applies to your research, read the appropriate section before selecting a response.

## Materials & experimental systems

| n/a | Involved in the study | |
|---|---|---|
| ☐ | ☒ | Antibodies |
| ☐ | ☒ | Eukaryotic cell lines |
| ☒ | ☐ | Palaeontology and archaeology |
| ☐ | ☒ | Animals and other organisms |
| ☒ | ☐ | Clinical data |
| ☒ | ☐ | Dual use research of concern |
| ☒ | ☐ | Plants |

## Methods

| n/a | Involved in the study | |
|---|---|---|
| ☒ | ☐ | ChIP-seq |
| ☐ | ☒ | Flow cytometry |
| ☒ | ☐ | MRI-based neuroimaging |

# Antibodies

| | |
|---|---|
| Antibodies used | Beckman Coulter: anti-V-PE/FITC kit (Beckman, IM3497); Biolegend: anti-CD3-APC (300312), anti-CD4-APC-Cy7 (300518), anti-CD8-PE-Cy7 (344750), anti-CD45-PerCP-Cy5.5 (368504), anti-HLA-A/B/C-Pacific Blue (311418), anti-HLA-DR/DP/DQ-PE (361716), anti-4-1BB-BV421 (309819), anti-CD25-PE-Cy7 (302612), anti-CD69-PerCP-Cy5.5 (310926), anti-CD45RA-AF700 (304119), anti-CD45RO-BV510 (304246), anti-GZMB-APC (372204), anti-IFN-PE (506507), and anti-TNF-APC-Cy7 (502944); Thermofisher: anti-human IgM-AF647 (314536) and anti-human IgG Fc-PE (MA110377). Protein L-PE (Cell Signaling Technology, #58036) and Lens Culinaris Agglutinin (LCA)-FITC (Thermofisher, L32475) staining were performed as surface antibody staining in 1xPBS. The antibody concentration applied in all assays adhere to the manufacturer's instructions and recommendations. |
| Validation | Primary antibodies were used for the target, species and application (flow cytometry) identified by the manufacturer. Research Resource Identifiers (RRIDs) were obtained from The Antibody Registry (antibodyregistry.org):<br>Biolegend 300312 RRID:AB_314048<br>Biolegend 300518 RRID:AB_314086<br>Biolegend 344750 RRID:AB_2687201<br>Biolegend 368504 RRID:AB_2566352<br>Biolegend 311418 RRID:AB_493669<br>Biolegend 361716 RRID:AB_2750318<br>Biolegend 309819 RRID:AB_10895902<br>Biolegend 302612 RRID:AB_314282<br>Biolegend 310926 RRID:AB_2074956<br>Biolegend 304119 RRID:AB_493762<br>Biolegend 304246 RRID:AB_2565801<br>Biolegend 345008 RRID:AB_11218598<br>Biolegend 329918 RRID:AB_2159324<br>Biolegend 372204 RRID:AB_2687028<br>Biolegend 506507 RRID:AB_315440<br>Biolegend 502944 RRID:AB_2562870<br>Biolegend 500326 RRID:AB_2125593 |

# Eukaryotic cell lines

Policy information about cell lines and Sex and Gender in Research

| | |
|---|---|
| Cell line source(s) | ThermoFisher: ExpiCHO; ATCC: Jurkat (TIB-152, Clone E6-1); Promega: Jurkat-NFAT-luciferase effector cells; |
| Authentication | none of the cell lines used were authenticated |
| Mycoplasma contamination | All cell lines tested negative for Mycoplasma contamination. |
| Commonly misidentified lines<br>(See ICLAC register) | No commonly misidentified cell lines were used in the study. |

# Animals and other research organisms

Policy information about studies involving animals; ARRIVE guidelines recommended for reporting animal research, and Sex and Gender in Research

| | |
|---|---|
| Laboratory animals | 8-12 wk old NOD.Cg-Prkdcscid Il2rgtm1Wjl/SzJ (NSG) mice were purchased from The Jackson Laboratory. Mice were bred and maintained under specific pathogen-free conditions with a 12 hr light/dark cycle at 70-72°F, 45-50% humidity with food and water provided ad libitum. |
| Wild animals | This study didn't involve wild animals |
| Reporting on sex | In this finding, we used both male and female mice |

Field-collected samples | No field collected samples were used in the study.

Ethics oversight | All of the in vivo studies were approved by the Yale University Institutional Animal Care and Use Committee. Mice were bred and maintained under specific pathogen-free conditions with food and water provided ad libitum. The Yale University animal facility is accredited by the Association for Assessment of Laboratory Animal Care.

Note that full information on the approval of the study protocol must also be provided in the manuscript.

# Plants

Seed stocks | No seed stocks were used in the study.

Novel plant genotypes | No novel plant genotypes were involved in the study.

Authentication | No authentication procedures were performed.

# Flow Cytometry

## Plots

Confirm that:

☒ The axis labels state the marker and fluorochrome used (e.g. CD4-FITC).

☒ The axis scales are clearly visible. Include numbers along axes only for bottom left plot of group (a 'group' is an analysis of identical markers).

☒ All plots are contour plots with outliers or pseudocolor plots.

☒ A numerical value for number of cells or percentage (with statistics) is provided.

## Methodology

Sample preparation | Cells were re-suspended in l00ul lxPBS containing FcR block (Biolegend, 422302)) and Aqua Live/Dead fixable dye (Thermofisher, L34957) for 10 minutes at RT. Without wash, l00ul antibody mixture in lxPBS was added for 30 minutes at 4° C. After washing in lxPBS containing 2% FBS, cells were re-suspended in 200ðl lxPBS containing 2% FBS and l0ul counting beads for flow cytometry detection were added. For intracellular staining, cells were fixed in 2% PFA for 20 minutes at RT and incubated with antibody mixture in lxPermeablization buffer (Thermofisher, 00833356) for 30 minutes at RT.

Instrument | Beckman CytoFLEX LX Flow cytometer

Software | CytExpert was used for data collection and FlowJo for data analysis.

Cell population abundance | Purity was determined by flow cytometry and reported in the Methods section.

Gating strategy | The gating strategy is provided in Figures and Supplementary Information.

☒ Tick this box to confirm that a figure exemplifying the gating strategy is provided in the Supplementary Information.
